# Risk factors for frequent unplanned hospital visits in older multimorbid patients after an emergency department visit: a retrospective cohort study

**DOI:** 10.1007/s40520-025-03280-5

**Published:** 2025-12-20

**Authors:** August Taegil, Anders Björkelund, Anne Ekdahl, Karol Biegus, Ulf Ekelund, Jonas Björk, Jakob Lundager Forberg

**Affiliations:** 1 Department of Geriatrics, Helsingborg Hospital, Helsingborg, Sweden; 2 Centre for Environmental and Climate Science, Lund University, Lund, Sweden; 3 Department of Clinical Sciences, Lund University, Lund, Sweden; 4 Department of Internal and Emergency Medicine, Skåne University Hospital, Lund, Sweden; 5 Department of Laboratory Medicine, Lund University, Lund, Sweden; 6 Forum South, Clinical Studies Sweden, Skåne University Hospital, Lund, Sweden; 7 Department of Emergency Medicine, Helsingborg Hospital, Helsingborg, Sweden

**Keywords:** Readmission, Multimorbid, Older patient, Emergency department

## Abstract

**Background:**

Hospital readmissions are common among older multimorbid patients, and hospitalisation is associated with functional decline and reduced quality of life. Identifying patient characteristics and risk factors for repeated hospital visits following an emergency department (ED) encounter is important for guiding targeted interventions to prevent future revisits.

**Aim:**

To characterise multimorbid older patients with frequent unplanned hospital visits following an emergency department (ED) visit, compare them to those with fewer revisits, and identify risk factors for frequent unplanned hospital revisits.

**Methods:**

A retrospective analysis of 25 436 multimorbid patients aged ≥ 75 years who visited 8 EDs in Sweden in 2017 was performed. Patients with ≥ 3 ED visits or unplanned hospital admissions in the following year were classified as frequent hospital visitors. Their characteristics were compared to those of infrequent visitors, and binomial logistic regression was used to identify factors predictive of frequent hospital visits.

**Results:**

An increasing number of hospital visits in the prior 12 months (odds ratio (OR) 1.25 95% confidence interval (95% CI) 1.21–1.29) and an increasing number of comorbidities (OR 1.11 95% CI 1.09–1.13) were the most impactful risk factors for multiple unplanned hospital revisits. Female sex (OR 0.81 95% CI 0.75–0.88) was the most important protective factor.

**Conclusion:**

A history of frequent hospital visits was the strongest risk factor for future unplanned hospital visits. Several other factors were also identified, suggesting the potential for predictive models to identify at-risk patients before frequent visits occur.

## Introduction

Readmission following hospitalisation is a common occurrence for older multimorbid patients, putting them at risk of adverse events such as falls [1] and delirium [2] in the short term and functional decline [1, 3, 4], loss of independence [3] and decreased quality of life [4] in the long term. Furthermore, as the population ages [5] and becomes increasingly multimorbid [6], readmission in this patient group contributes to a growing challenge with crowding in healthcare systems [7]. Therefore, reducing readmission in older multimorbid patients is an important clinical, policy, and research goal [8–12].

Fortunately, previous research has indicated that some hospital readmissions are avoidable through interventions such as better care planning and post discharge follow-up [11–13]. Thus, an important task is to identify patients at risk of readmission to target effective interventions [12]. Despite readmissions being extensively studied [14, 15], the prediction of who is actually at risk of readmission is challenging [16, 17]. Older multimorbid patients are typically defined as those aged 60 or 65 years and above [14, 18], but few studies have focused specifically on patients aged 75 years or older [1]. This is problematic, as increasing age is associated with more complex and increasing health issues, which alter healthcare needs and utilisation [19–24], and this group may be more clinically relevant for preventive interventions.

Furthermore, most available research does not follow patients across different healthcare providers [14, 15] or beyond the typical 30 days after hospital discharge [14]. Identifying risk factors over a longer period, such as a 12-month follow-up, may be key to recognising patients whose trajectories could be altered through targeted interventions. The ED is an important early contact point in healthcare systems [25], where indicators for future unplanned hospital visits, that is unplanned hospital admissions and future ED visits, may first appear. Thus, exploring patient characteristics at the ED level is vital for the development of effective preventive strategies.

The aim of this study was to characterise multimorbid older patients (aged 75 years or older) who are frequent unplanned hospital visitors in the year following an ED visit, compare them to those with fewer unplanned revisits, and finally identify risk factors associated with frequent unplanned hospital visits.

## Methods

### Settings and data sources

The Skåne Emergency Medicine (SEM) cohort [26] is a database containing comprehensive data on adults visiting EDs in the region of Scania in southern Sweden. The database spans the period of January 1st, 2017– December 31st, 2019. The ED cohort included all visits except those to dedicated psychiatric, gynaecology, and ophthalmology EDs. The dataset covers information on ED visits, hospital admissions, diagnoses in secondary and tertiary care, mortality and causes of death. In addition to medical information, the SEM also incorporates individual-, household-, and area-level sociodemographic data.

The data sources for SEM include both regional databases, such as electronic healthcare records, and national Swedish registers and databases. Swedish identification numbers, a unique number for each Swedish citizen and registered resident, were used for data linkage between registers and databases. The formation of the SEM cohort and its data sources are described in detail elsewhere [26].

## Definitions and selection of patients

**Fig. 1:**
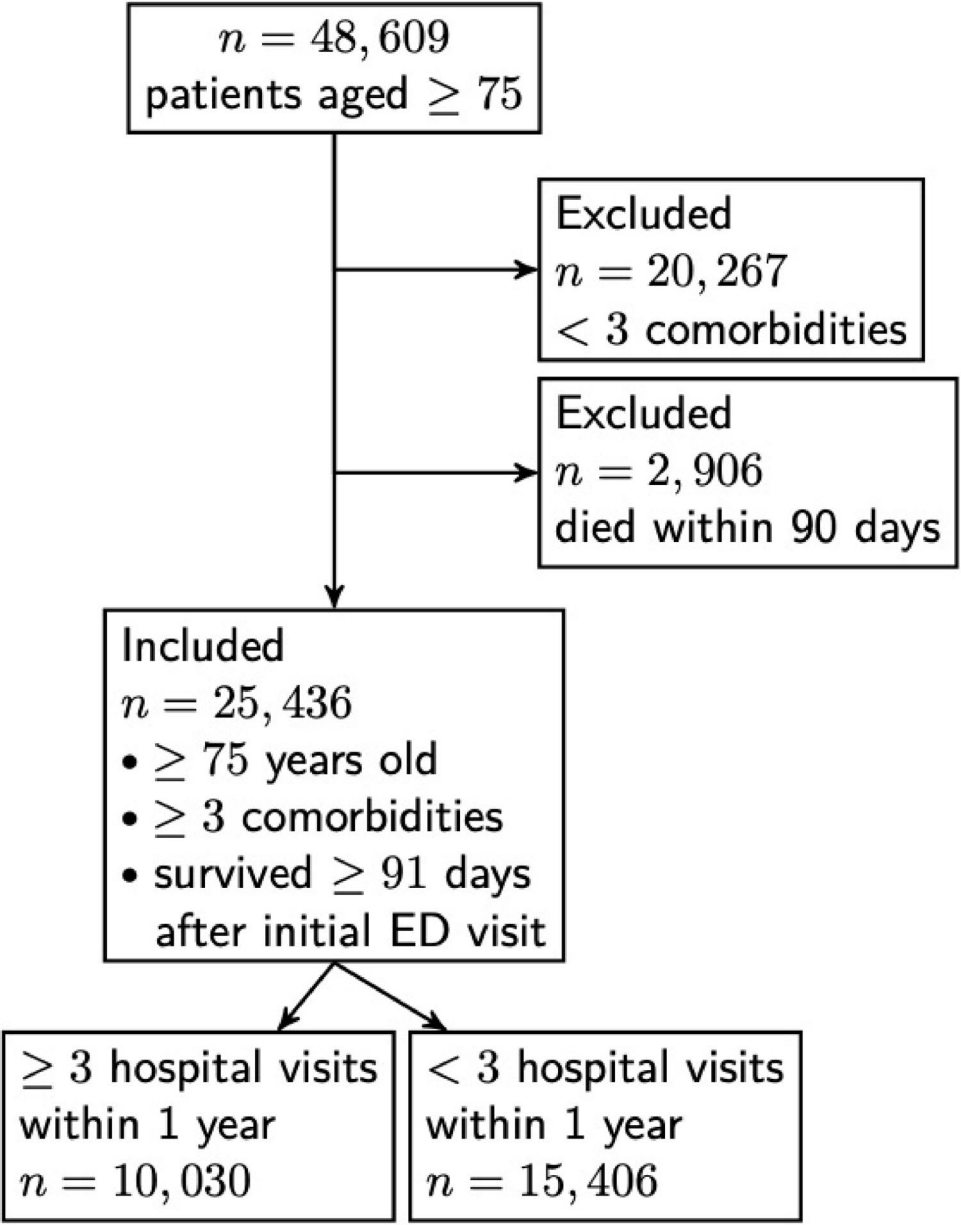
Patient attrition and classification into frequent unplanned hospital visitors and infrequent unplanned hospital visitors

In this study, the intention was to investigate frequent unplanned hospital visitors among older multimorbid patients presenting at the ED. We therefore began by defining this patient group as well as a comparison group of infrequent unplanned hospital visitors in the same age bracket visiting the same EDs. In Sweden, multimorbid older patients are defined on the basis of a combination of age, comorbidity count and healthcare usage [8]. Consequently, SEM patients were considered older and multimorbid if they were aged ≥ 75 years at the time of the index ED presentation and had received diagnoses from ≥ 3 different ICD-10 chapters within the preceding five years. Among these, patients who also had ≥ 3 unplanned hospital admissions or ED visits (including the index ED visit) within 12 months of the initial ED visit were classified as frequent hospital visitors (FREQs). Patients who only had a total of 1–2 unplanned hospital visits (including the initial ED visit) were classified as infrequent hospital visitors (INFREQs). Only patients who presented at an ED in 2017 were included to ensure that all patients had at least one year of follow-up. Patients who died within 90 days of their initial ED visit were excluded, as potential interventions for these patients likely focus on palliative initiatives rather than preventing readmissions. Patient selection and FREQ and INFREQ group allocation are shown in Fig. 1.

### Statistical analysis

For the univariable analysis, chi2 tests were used to compare between-group proportions, and independent samples t tests were used for comparison of means. Binomial logistic regression with backward selection was used for the multivariable analysis. Patients who had variables with missing data were excluded from the binomial logistic regression model. Statistical significance was set at *p* < 0.05 for all tests. All the statistical analyses were performed via SPSS Statistics version 29.0.2.0.

## Results

Among the 48 609 individual patients who were 75 years or older in the SEM cohort, 25 436 (52.3%) patients had both received a diagnosis from ≥ 3 different ICD-10 chapters in the last five years and survived at least 91 days following the initial ED visit. Of these patients, 15 406 were categorised as INFREQ and 10 030 were categorised as FREQ (Fig. 1).

## Univariate analysis

For the sociodemographic data, the FREQs were statistically significantly older, less educated and more likely to live in more socioeconomically affluent areas than the INFREQs. The proportion of females and the proportion of patients married/having a registered partner were greater in the INFREQ (Table 1). No statistically significant differences were found between the FREQ and INFREQ in terms of income from pensions, the proportion of patients living rurally, or the proportion of patients born outside of Sweden (Table 1).

**Table 1:** Sociodemographic characteristics of older multimorbid ED patients with three or more versus fewer than three unplanned hospital visits within one year

Factors	Infrequent hospital visitors	Frequent hospital visitors	*p* value
Number of patients	15 406	10 030	
Age, years, Mean	82.6	82.9	**< 0.001**
Females, Mean	83.1	83.4	**0.026**
Males, Mean	81.9	82.4	**< 0.001**
Age ≥ 85, %	35.1	37.7	**< 0.001**
Age ≥ 90, %	13.2	13.9	0.128
Age ≥ 95, %	2.8	2.5	0.121
Sex female, %	57.9	53.6	**< 0.001**
Married/registered partner vs. not, %	43.8	41.5	**< 0.001**
Income from pension (SEK/year), Mean	141 299.2	142 143.7	0.179
Attended upper secondary school, %	40.7	38.2	**< 0.001**
Socioeconomic index^a^ for patients’ area of residence, Mean	10.4	10.0	**< 0.001**
Area type rural, %	9.6	9.7	0.871
Born abroad, %	12.8	13.2	0.286

^a^ Socioeconomic index from Statistics Sweden, representing the socioeconomic conditions in the area the patient lives in. The values of the index ranges from 0 to 100, where 0 represents the score with the best socioeconomic circumstances and 100 represents the worst socioeconomic circumstances

In terms of medical characteristics at the initial ED visit, compared with the INFREQ, the FREQ had significantly more comorbidities, medications and hospital visits in the prior 12 months. Furthermore, the FREQ scored significantly higher on the Charlson Comorbidity Index and had more abnormal laboratory values. Compared with the INFREQ patients, the FREQ patients were also more likely to undergo imaging at the initial ED visit and be transported to the ED with an ambulance (Table 2). In terms of patient outcomes, FREQ patients were significantly more likely to be admitted to the hospital at the initial ED visit, had more hospitalisations in the following 12 months, and had higher mortality than INFREQ patients did (Table 2).

**Table 2:** Patient characteristics and outcomes of older multimorbid ED patients with three or more versus fewer than three unplanned hospital visits within one year

Factors	Infrequent hospital visitors	Frequent hospital visitors	*p* value
Known medical characteristics at the initial ED visit			
Number of comorbidities, Mean	5.1	5.8	**< 0.001**
Charlson Comorbidity Index	1.7	2.4	**< 0.001**
Number of medications, Mean	7.4	8.5	**< 0.001**
Hospitalisations 12 months prior 1 ≥, %	27.5	40.6	**< 0.001**
Number of hospitalisations, Mean	1.5	1.8	**< 0.001**
Days in hospital prior 12 months, Mean	10.9	14.5	**< 0.001**
In hospital surgery 1–30 days prior to the initial ED visit, %	2.0	1.9	0.469
Ambulance transport to the ED, %	44.3	46.9	**< 0.001**
Number of abnormal laboratory values^a^, Mean	3.1	3.4	**< 0.001**
Patients with hyponatremia/patients tested	2 435/11 404	1 873/7 946	**< 0.001**
Patients with elevated creatinine/patients tested	4 850/11 425	3 857/7 956	**< 0.001**
Patients with anaemia/patients tested	3 552/11 414	3 074/7 958	**< 0.001**
Any imaging at the initial ED visit, %	53.7	57.7	**< 0.001**
Outcome following the initial ED visit			
Admitted at index, %	41.9	45.8	**< 0.001**
Days spent in hospital at index, Mean	7.6	8.1	**0.004**
Hospital visits following 12 months, Mean	0.4	3.6	**< 0.001**
Days spent in hospital following 12 months, Mean	5.6	20.4	**< 0.001**
Days/hospital stay following 12 months, Mean	7.7	8.4	**< 0.001**
Deaths in the study period (2017–2019), %	22.0	41.4	**< 0.001**
Days from initial ED visit to death, Mean	502.6	449.5	**< 0.001**

^a^ Available laboratory data on: Plasma C reactive peptide, Plasma troponin t, Plasma-sodium, Plasma-creatinine, Plasma-ionised calcium, Plasma-glucose, Plasma-lactate, Plasma-leucocytes, venous pH, Blood-Haemoglobin.

### Multivariate analysis

In the binomial logistic regression, FREQ was chosen as the dependent variable. Five-hundred and twenty-three out of the 25 436 patients had missing data in at least one variable and were consequently excluded from the regression model. After adjusting for the other independent variables, the following factors were associated with an increased risk of FREQ: increasing number of hospital visits in the previous 12 months (OR 1.25), increasing number of comorbidities (OR 1.11), increasing number of abnormal laboratory findings at index (OR 1.05), increasing number of medications (OR 1.04 and increasing age (OR 1.01). Factors associated with a decreased risk of FREQ were: female sex (OR 0.81), being married/having a registered partner (OR 0.90), having attended upper secondary school (OR 0.93) and ambulance transport to the initial ED visit (OR 0.94). The statistically significant independent variables are summarised in Table 3. The nonsignificant variables removed from the model included income from pensions, socioeconomic conditions in patients’ area of residence, rural residential area, being born outside of Sweden, and admission at the index.

**Table 3:** Risk factors for frequent unplanned hospital visits, defined as three or more ED visits and/or hospital admissions within one year following the initial ED visit

Factors	Odds ratio	95% confidence interval
Age, years increasing	1.01	1.00–1.01
Female sex	0.81	0.75–0.88
Married/registered partner vs. not	0.90	0.85–0.95
Attended upper secondary school vs. not	0.93	0.88–0.98
Number of comorbidities, increasing	1.11	1.10–1.13
Number of medications, increasing	1.04	1.03–1.05
Number of hospital visits in the previous 12 months	1.25	1.21–1.29
Ambulance transport to the ED	0.94	0.89–1.00
Number of abnormal laboratory findings at index, increasing	1.05	1.04–1.07

## Discussion

In this study, we analysed the characteristics of older multimorbid ED patients with and without frequent hospital visits. A greater number of hospital visits in the prior year was the strongest risk factor for frequent future hospital visits. Several other differences were identified, but no single characteristic clearly distinguished frequent and infrequent hospital visitors.

The frequent hospital visitors’ relatively worse clinical outcomes were reflected in the medical data. They had a higher mean number of medications, comorbidities and hospital visits in the prior 12 months. These are all previously identified risk factors for readmission [14, 15, 27–31]. Interestingly, despite it being slightly more common for the FREQ patients to have had ambulance transport to the ED (see Table 1) ambulance transport was slightly protective against being a frequent hospital visitor when adjusting for other factors in the multivariate analysis (OR 0.94, 95% CI 0.89–0.997).

The sociodemographic data did not show a clear pattern distinguishing FREQ from INFREQ. FREQ patients had a lower education level as a group, but income and residence area-level socioeconomic deprivation were not associated with increased readmission risk. While many studies have shown that lower socioeconomic status, including a lower education level [27], lower income [29], and living in deprived areas [32], is associated with higher readmission rates, others have reported no such associations [15]. In our study, being married or having a registered partner was a protective factor against future frequent hospital visits. Mixed findings exist for associations between marital status and hospital readmissions [18, 27, 30, 33]. In line with previous findings, male sex was associated with a greater risk of future hospital visits [14, 29–31]. The associations between age and the risk of readmission are inconsistent in the literature. Several studies, such as ours, have reported a positive correlation [29, 31], whereas others have reported no association [27, 28] or even a protective effect at higher ages, possibly due to a preference for home treatment in patients receiving adequate home health and social care [34]. Our data lacked information on home health and social care or residential status, which prevented us from investigating whether these factors influenced the risk of readmissions in our cohort. Migration status and rural residence did not differ between frequent and infrequent hospital visitors. In contrast, some studies have reported increased readmission risk for ethnical minorities [32, 35, 36]. Similarly, rural residence showed no association in our study, which is consistent with the findings of other studies [27, 32].

This study contributes to our knowledge of hospital admissions in older multimorbid patients in two important ways. First, few studies have focused specifically on readmissions in patients aged 75 years and older [14]. Older multimorbid patients are typically defined as those aged 60 or 65 years and above [14, 18] with two or more chronic conditions [18]. However, this definition covers a large and heterogeneous group [37], representing between 55% and 98% of all older individuals [38, 39]. Patients aged 75 years and older differ in morbidity and healthcare use compared with this broader group [19–24], highlighting the need for targeted data to better predict readmissions in this population. This need is underscored by the growing number of patients in this age group [5, 9]. Second, this study adds to knowledge on both short- and long-term risks of readmission. While early readmission within 30 days of discharge may be reduced though interventions such as pharmacist reviews [40] and improved discharge planning [12], evidence for reducing readmissions over longer time periods is limited. Neither hospital-at-home services nor mobile medical units have reliably shown overall benefit in reducing long-term readmissions risk [41, 42], although they may be effective for certain subgroups of older multimorbid patients [42, 43]. As most studies on readmissions focus on early readmission [14, 30, 31], long-term risks remain relatively unexplored. More studies, such as ours, particularly those that also include data on frailty and functional status, could improve risk identification and support efforts to target older patients who may benefit from preventive interventions to reduce hospital reattendance.

A strength of our study was the use of high-quality data from both regional and national sources, minimising missing data. Data linkage enabled us to distinguish between planned and unplanned hospital admissions, reducing confounding from nonemergent care. Our study also had several limitations. First, as a retrospective study, no causal relationships could be concluded. While our data sources were based on healthcare records and registers, which may introduce information bias, the large cohort size supports the validity of our findings. Some limitations within the dataset exist. For example, the data are now a few years old and were collected before the COVID-19 pandemic. Although this may imply some changes in demographics and healthcare provision, we believe these differences are minor in our setting and do not affect the overall findings. Another limitation with the data source is the lack of data from primary care and the reliance only on secondary and tertiary care records. This likely leads to a slight underestimation of multimorbidity, which could affect the generalisability of the results. We also lacked national data on ED visits outside our region. However, the inclusion of national hospital admission data and the low likelihood of this population seeking care outside the region likely minimised misclassification between the FREQ and INFREQ groups. Perhaps the most important limitation is the lack of data on key features such as frailty [14], functional and [14, 31, 44], cognitive status [14], municipal care services [31] and social circumstances [15]. Including this data might have better distinguished patients at high versus low risk of frequent hospital visits.

Our study differs from most previous research by focusing on the risk of multiple unplanned hospital contacts rather than a single event. We deliberately selected a population with more complex morbidity patterns than those included in many earlier studies to better understand high healthcare users among older multimorbid patients. This complexity increases the potential for confounding in general, and for confounding by indication in particular, which we sought to mitigate through multivariable binomial logistic regression allowing adjustment for several independent factors. However, unmeasured variables, particularly frailty and functional status, were not available, and residual confounding may therefore remain. Another way our design differed from many other studies was that we also included ED visits without subsequent hospital admission as an outcome. While this makes comparisons with other studies more challenging, it offers some advantages: it better reflects the experience of high-use multimorbid patients, provides more relevant data for predictive models and preventive strategies, and aligns with the reality of fewer available hospital beds per capita [45], which often results in prolonged ED assessments without subsequent admission. Another way in which our study stands out is the exclusion of patients who died within 90 days of the index ED visit. The rationale for this was that patients who died within 90 days were likely to have clinical needs related to palliative care interventions. In contrast, the patients who survived longer – and consequently were included in the study – were likely to benefit from interventions aimed at reducing hospital revisits. Still, some survival bias may persist in this group as it is likely that some patients who died soon after the 90-day cut off were classified as INFREQ solely because their time at risk for repeat hospital contacts was truncated by death, and these patients may otherwise have fulfilled the criteria for the FREQ group. However, as our focus was to estimate factors leading to frequent unplanned hospital visits, attempts to account for competing risk – such as death – would have generated a somewhat artificial population with estimates partly reflecting unplanned hospital needs had death not occurred. For this reason, we were reluctant to account for competing risks as it was not obvious that it would have increased the clinical validity of the findings.

We found several statistically significant differences between FREQ and INFREQ via the available data. Some significant differences likely reflect the large sample size and not all are necessarily clinically meaningful, such as a three-month difference in age or a small difference in annual income. Indeed, some of these changes are not even going to be discernible to physicians in the ED. However, the differences may still be valuable for machine learning tools designed to assist in ED decision-making [17, 46], particularly as readmission prediction models have become increasingly accurate in recent years [16]. A recent systematic review by Askar et al. demonstrated that diverse data types—including demographics, diagnoses, and laboratory values—can be used effectively to predict hospitalisation [17]. We anticipate that ongoing advances in machine learning, together with large high-quality datasets, will further improve the ability to identify patients at high risk of readmission. When this information is applied in implementing interventions that specifically target certain patient groups, hospital readmissions could be more effectively reduced [12]. However, as these interventions are often expensive and time-consuming [12, 41, 42], the scarcity of resources in healthcare systems has to be considered. Preventative interventions should therefore be targeted only to patients for whom they are proven to be effective and who are at high risk of future hospital readmissions [12].

## Conclusion

A history of prior hospital visits was the strongest independent risk factor for future unplanned hospital contacts among multimorbid older patients. Additionally, weaker risk factors across clinical and sociodemographic domains were also observed. While no single factor was sufficient for accurate identification, combining multiple moderate risk factors in multifactorial models that include frailty and functional status may improve early risk detection.

## Data Availability

Anonymized parts of the SEM database will be available for sharing on reasonable request. Please send an email to ulf.ekelund@med.lu.se.

## Abbreviations

ED: Emergency department
OR: Odds ratio
CI: Confidence intervals
FREQ: frequent hospital visitors
INFREQ: Infrequent hospital visitors
SEM cohort: Skåne Emergency Medicine cohort
ICD-10: International Classification of Disease 10th version
SEK: Swedish kronor
